# A Research-Practice Partnership to Develop the R-CITY Multi-Component, Equity-Focused Social–Emotional Learning Intervention

**DOI:** 10.1007/s12310-024-09703-4

**Published:** 2024-08-30

**Authors:** Jessika H. Bottiani, Maisha Gillins, Charity Brown Griffin, Chelsea A. Kaihoi, Lorenzo Hughes, Sharon Pendergrass, Toshna Pandey, Ryan Voegtlin, Sandy Rouiller, Elise T. Pas, Katrina J. Debnam, Catherine P. Bradshaw

**Affiliations:** 1 School of Education and Human Development, University of Virginia, Charlottesville, VA USA; 2 Anne Arundel County Public Schools, Office of Equity, Annapolis, MD USA; 3 Psychological Sciences, Winston-Salem State University, Winston-Salem, NC USA; 4 Department of Student Services, Anne Arundel County Public Schools, Annapolis, MD USA; 5 Sheppard Pratt, Baltimore, MD USA; 6 Johns Hopkins Bloomberg School of Public Health, Baltimore, MD USA

**Keywords:** Research-practice partnerships, Equity, Social emotional-learning, Universal interventions

## Abstract

There is growing interest in the integration of social–emotional learning (SEL) and equity approaches in schools, yet systematic research on how to blend these two frameworks is limited. In this article, we describe the process by which a research-practice partnership (RPP) collaborated to iteratively co-create a multi-component equity-focused SEL preventive intervention in the context of a politically charged landscape related to the ‘dual pandemics’ of racial injustice and COVID-19 in the early 2020s. We conducted a document review of informal data sources (e.g., meeting minutes, correspondence) and analyses of formal data sources (i.e., teacher interviews, student focus groups) to describe how we overcame challenges to form an RPP, to demonstrate our collaborative intervention development efforts, and to assess feedback on the contextual appropriateness of the intervention. We discuss lessons learned from our partnership efforts and reflect on future directions for RPP-driven work to advance equity-focused SEL in K-12 public schools.

High rates of discrimination and bias in schools negatively impact students’ mental health, safety, wellbeing, and learning (La Salle-Finley, 2023; Leath et al., 2019). Yet there are relatively few school-based mental health or social and emotional learning (SEL) interventions focused on fostering adult and student skills to reduce bias and promote equity in schools (Gregory et al., 2021; Jennings et al., 2021; Jones et al., 2021). Although the development of such interventions should be a central focus as schools recover from the COVID-19 pandemic, such work has been especially challenged by the politically charged context related to equity and SEL in the early 2020s. Meaningful engagement of students, teachers, and decision-makers is necessary to ensure the contextual appropriateness, feasibility, acceptability, and uptake of these interventions. This study fills a gap in the literature by describing the formation and collaboration of a university and school district research-practice partnership to develop an intervention that blended equity and SEL. Specifically, this paper describes the process by which the partnership collaboratively developed the *Reducing Racism and Violence through Collaborative Intervention with Teachers and Youth* (R-CITY) program, a multi-component, equity-focused SEL intervention that aimed to prevent violence by mitigating racism and other forms of bias and discrimination in schools.

## Bias Experiences and Student Mental Health in the Peri-COVID Recovery Period

As concerns about youth mental health escalated during the COVID-19 recovery period of the early 2020s, public alarm about bias and discrimination and its impact on young people also erupted, following persistent incidents of hate speech occurring in schools. In 2023, bias incidents in the news included student fans chanting racial slurs during sports games (Swanson, 2023), graffiti of hate symbols like swastikas on school property (Griffin, 2023), and students posting selfies in blackface on social media (Reuille, 2023). Data suggest these incidents are on the rise. A district in Maryland reported one hate-based incident per day on average in 2022, tripling the rate of the prior year, with a majority of these incidents being antisemitic. State and national data similarly suggest that anti-Black, antisemitic, anti-Muslim, anti-Sikh, and anti-LGBTQIA+bias incidents are on the rise in many regions of the United States (U.S.), frequently occurring in schools (Federal Bureau of Investigation, 2023; New Jersey Attorney General’s Office, 2023; U.S. Department of Justice, 2023).

Research has long documented the harmful effects of exposure to hate speech and discrimination on the mental health and healthy development of marginalized students (LaSalle-Finley et al., 2023), such as students who are Black, Latine, Indigenous, immigrant, religious minorities, LGBTQIA+, or living on a low income. Experiencing microaggressions, or other more overt forms of bias, impedes efforts of marginalized youth to learn (Leath et al., 2019). Specifically, such everyday forms of discrimination communicate to students that they are inferior or not a part of the school community, which undermines young people’s ability to feel a sense of belonging at school, a core psychological need and precursor to student engagement (Eccles & Roeser, 2011). Structural forms of discrimination, as demonstrated by disproportionate use of exclusionary discipline with Black and Brown youth, also suggest to students that they are not accepted or do not fit in (Stevenson, 2014; Voight et al., 2015).

## Social–Emotional Learning and Preventing Bias

School-based preventive interventions play a critical role in supporting student mental health (Evans et al., 2023), particularly in the peri-COVID-19 recovery period (Schwartz et al., 2021; Weist et al., 2024). Yet, despite impacts of discrimination on youth mental health, most school-based preventive interventions have not focused on addressing bias (Jones et al., 2021). Traditional social and emotional learning curricula aim to promote more positive social environments for students and teach core competencies, including Self-Awareness, Social Awareness, Responsible Decision-Making, Self-Management, and Relationship Skills (Collaborative for Academic, Social, & Emotional Learning [CASEL], 2015). In some instances, curricula include concepts related to prejudice (e.g., see *Second Step* curriculum; Frey et al., 2000). However, most curricula do not address how to apply SEL skills comprehensively to the complex circumstances that arise in the context of bias incidents, nor do they teach systemic equity-specific concepts (e.g., intersectionality, positionality). Traditional SEL programs are also not typically designed for or with marginalized students (e.g., see Henderson Smith et al., 2022), or with the specific goal of promoting their experiences of equitable, inclusive, and just school social environments (Gregory et al., 2021). This is a missed opportunity, as SEL skills could be taught in a way that prevents exposure to identity-based discrimination at school.

Transformative SEL is a framework that extends traditional SEL to feature “equity elaborations” on SEL competencies (Jagers et al., 2018, p.3). The framework encourages an SEL instructional focus on creating equity-relevant skills within CASEL’s broader five competency domains. The equity-focused competencies include *identity* (Self-Awareness), *belonging* (Social Awareness), *curiosity* (Responsible Decision-Making), *agency* (Self-Management), and *collaborative problem-solving* (Relationships Skills). The transformative SEL framework holds promise to address critiques that SEL is too traditionally focused on White values and competencies (Simmons, 2021) and may help center student equity in discussions about specific practices for implementing SEL programming in schools. Yet interventions addressing transformative SEL competencies are only emerging (e.g., Pas et al., 2024; Umaña-Taylor et al., 2024). For example, some content in the high school *Well Connected©* curriculum explicitly teaches students about understanding their *identity*, building a sense of *agency* to address social justice issues, and having a mindfulness practice (aligning with *curiosity*; see Pas et al., 2024). Knowledge of these concepts may provide a foundation for the prevention of bias and discrimination at interpersonal levels and beyond. However, more university-district partnered research is needed to develop and assess feasibility and acceptability of such intervention content.

## Need for University-District Research-Practice Partnerships to Advance Educational Equity

Education-focused research-practice partnerships have been defined as long-term, reciprocally beneficial, intentional relationships between researchers and district leaders to address challenges that the partner district identifies as pressing, rather than researcher identified gaps in theory or knowledge (Coburn et al., 2013). Over the years, research-practice partnerships (RPPs) have been embraced as a promising approach to resolve critical problems of practice in education, democratize participation in the generation of research evidence, and support diffusion of effective practices (Sjölund et al., 2022). Features of effective RPPs have been theorized to include the importance of building trust, focusing on research that informs action, and supporting the partner practice organization in achieving its goals (Henrick et al., 2017). However, despite a robust body of literature on participatory research and RPPs (Coburn et al., 2016; Farrell et al., 2022), practices for initiating and sustaining partnerships to advance equity-focused SEL in schools are understudied.

Shifts in the sociopolitical climate around equity and SEL in the early 2020s made school-based implementation of equity-focused SEL particularly contentious. Engaging partners at both district- and school-levels is vital to garner needed buy-in at multiple levels, integrate with other ongoing initiatives, and ensure implementation supports are embedded so that interventions are seen as feasible and acceptable by implementers (Bradshaw & Haynes, 2012). In this politically charged context, an RPP between a university and district can help balance these needs and sensitivities when developing and implementing an equity-focused SEL intervention in schools. A foundation of partnership, close collaboration, and meaningful engagement of students, parents, teachers, and decision-makers may help to ensure the contextual appropriateness of such interventions. For example, such partnerships may help to identify specific contextual considerations needed to respond to evolving restrictions on what teachers can say about race, gender, and sexuality. Yet more research is needed to illustrate how to establish and sustain equity-focused RPPs, as well as to demonstrate their utility in navigating pushback on equity and SEL in ways that are acceptable and feasible, to advance the work of equity in schools.

## Current Study

The purpose of this article is to describe a university-district RPP that iteratively co-created and implemented a developmentally tailored (i.e., elementary and middle school), multi-component equity-focused SEL preventive intervention. Specifically, in alignment with this special issue “Systematic Research on University-Community Partnerships for the Development of School Mental Health Interventions” in *School Mental Health*, this article has three goals. The first goal is to illustrate how we overcame challenges in establishing an RPP to develop an equity-focused SEL program during the dual pandemic of COVID-19 and racial inequity in the early 2020s, drawing on data from research team correspondence with districts and research team meeting minutes. The second goal is to demonstrate how the RPP collaborated to co-develop the content of the lessons, engagement strategies, and implementation supports, leveraging data from RPP meeting agendas and minutes, correspondence, and research team coach notes. The third goal is to assess the contextual appropriateness, feasibility, and acceptability of the RPP-developed program content and supports through our analysis of qualitative data collected in teacher semi-structured “exit” interviews and student focus groups. We conclude with a reflection on key lessons learned. This article addresses a gap in the literature on how university-district RPPs can collaborate to develop and implement equity-focused SEL interventions to reduce exposure to bias and discrimination at schools. We hope the article will inform future efforts to build and sustain successful university-district RPPs working toward educational equity.

## Method

### Research Ethics

The project was approved by the universities’ Institutional Review Boards, as well as the district’s research office. Teacher and student participants provided informed consent.

### Context

The RPP efforts described in this article took place within a large school district of about 85,000 economically, geographically, and racially diverse students in approximately 125 urban, suburban, and rural public schools in a Mid-Atlantic state. The student population in this district was 49% White, 22% Black; 19% Hispanic, 6% multi-racial/ethnic, 4% Asian, and < 0.05% American Indian or Alaska Native. The partnering researchers were from a school of education and human development at an R1 public research university in an adjacent state and a historically Black college/university in another neighboring state.

### District and University Partners’ Positionality

District personnel who were engaged in this project included representatives from the district’s designated equity office. This office was staffed by a chief equity officer, a senior manager for equity, three equity specialists, two family and community liaisons, and a student intern. Equity office staff identified as African American (63%), White (12%) and Latino (25%) and were two cisgender men and six cisgender women ranging in age from late teens to their 50s. The primary partners were the chief equity officer and the senior manager for equity; in addition, equity specialists were engaged at various points to ensure alignment of intervention content and activities with the vision of the equity office. The chief equity officer and senior manager for equity were former middle and high school teachers, middle and high school assistant principals, and principals, and both held central office administrative roles. The three equity specialists were former middle and high school teachers. The district’s department of student services also was involved in implementation, with representation from the director.

Partnering university faculty included a research associate professor, who was a White woman with roughly a decade of experience in culturally responsive, collaborative prevention science, and an associate professor and researcher at a historically Black university, who was a Black woman trained as a school psychologist. Her work collecting the stories of racially marginalized youth and the challenges and hopes they find in the U.S. education system guided her integration of a student-centered perspective into the RPP. Also engaged in the project were four coaches who were subcontractors of the university. Coaches identified as African American (50%), White (25%), and ethnically mixed Latino and White (25%) and were two cisgender men and two cisgender women ranging in age from their 20s to their 50s. One coach was a former teacher and designated as the lead coach given her subsequent expert training and extensive experience (over a decade) in motivational interviewing, a key component of the coaching framework utilized in this study. The other three coaches were highly skilled clinicians (degrees, professional training, and experience in clinical psychology and social work), two of whom had extensive experience working therapeutically with African American urban youth in research- and practice-focused contexts, and one of whom was also a former teacher.

### Data Sources and Analytic Approach

#### Goal 1 and Goal 2: Document Collation and Review

The first goal of this paper was to illustrate how challenges were overcome in establishing the RPP, and the second goal of this paper was to demonstrate how the RPP collaborated to co-develop the intervention. To accomplish these two goals, we drew upon various documents to construct a cohesive picture of the activities of the RPP and key decisions made collaboratively during the intervention co-development process. Data sources included university partner internal meeting agendas and minutes, RPP (i.e., university and district partner combined) meeting agendas and minutes, email correspondence, Outlook calendar invitations, shared Google Drive materials with embedded comments, and coach implementation notes.

The research partner’s project director collated documentation en masse. She reviewed the data sources and generated an initial timeline of key events, decisions, and actions that occurred, while engaging in a reflexive process to develop inferences. Thereafter, the practice partner organization reviewed the initial draft and provided written edits. In addition, through two working meetings (approximately 3 h total), the RPP collaboratively reviewed, adjusted, extended, and finalized inferences reported in the Results.

#### Goal 3: Qualitative Data and Analyses

The third goal of the paper was to assess the contextual appropriateness, acceptability, and feasibility of the intervention from the perspective of students and teachers. To accomplish this goal, we leveraged data from teacher semi-structured “exit” interviews and student focus groups. We describe the participants, data collection tools, and analyses for these data sources separately below.

##### Teacher Semi-Structured “Exit” Interviews

Teacher participants were a subset of 16 teachers who taught 4th through 8th grades and implemented the equity-focused SEL lessons. They were 87% cisgender female, and none identified as transgender, non-binary, or another gender identity. About 44% identified as White, 38% Black, and 19% another race or multi-racia. Teacher participants varied in age (most common age band 31–40 years) and experience (average: 11 years; range: < 1 year–24 years). Semi-structured interviews were an average length of 45–60 min and were conducted via video conference with participants from the first year of implementation (*n* = 2 teachers), whereas after COVID-19 pandemic restrictions were lifted, they were conducted in-person by coaches with participants from the second year of implementation (*n* = 14 teachers). The questions asked included: What was your experience with the equity lessons (e.g., most/least helpful, areas for improvement, [dis]comfort teaching the content)?; In creating a safe space and community for the implementation of the lessons (equity-focused SEL curriculum), how were you able to recognize and honor different identities in your classroom? How were you able to hold space for vulnerable conversations and topics?; How was your experience with the coaching and how would you change it to make it better (e.g., what was most/least helpful and comfortable?); What implementation supports for teachers do you think were or would be most helpful (e.g., demonstrating implementation, professional development sessions, online modules, coaching)? Teacher participants were compensated $50.

Teacher interview audio recordings were transcribed by a web-based transcription service and proofread by research assistants. All identifiable information was removed from the transcripts, and each was assigned a pseudonym and entered into Dedoose, a qualitative data coding software. A codebook with codes anticipated to emerge from the interviews was created using qualitative content analysis, which allows phenomena to be described by systematically coding and classifying data to reveal patterns (Maier, 2017; Schreier, 2012). Three research assistants were trained to conduct the coding: one primary coder and two secondary coders. The primary coder coded all the transcripts, and in a second round, secondary coders each coded about half of the transcripts. To address reliability, a co-author on this paper (not a primary or secondary coder) reviewed and cross-referenced all excerpts and codes, and when there were discrepancies, served as a tie-breaker to reconcile them. The coded data were then extracted and used as anchors to create the categories (successes, challenges, and suggested improvements) that are presented in the results.

##### Student Focus Groups

Students participated in focus groups and were recruited from the district’s Student Equity Advisory Team (SEAT) with support from district personnel. The SEAT was a representative group of students (Grades 8–12) empowered as part of the district’s commitment to ensuring that students ‘have a seat at the table.’ A purposeful sampling strategy (Palinkas et al., 2015) was used and students were eligible if they were in grades 4–9 and identified as a member of a racially marginalized group due to historical and contemporary experiences with inequity. The final sample included 15 participants from three focus groups in grades 4–9 (M_age_ = 12.3; 10 girls and 5 boys). Age stratification was used so that participants similar in developmental stage were grouped together to support describing their developmentally specific perceptions of the lessons. The semi-structured focus group sessions averaged 90 min and were held virtually via Zoom. The protocol exposed participants to the lessons and asked open-ended questions to elicit information about their perceptions of the lessons (e.g., What is your favorite part of the lesson? What part of the lesson would you change?). Student participants were compensated $25.

Student focus group data were automatically transcribed, and a triple-checking procedure was followed. Student data were compiled and analyzed within Delve qualitative data analysis software by one of the partnering university faculty. She read and reread all the focus group transcripts and created codes, noting emerging themes (Creswell & Creswell, 2017). These codes and categories were compared, contrasted (between and within focus groups), and sorted until no new categories and codes emerged and thematic saturation was reached (Creswell & Creswell, 2017). Bracketing and peer review were used to mitigate researcher bias and enhance the trustworthiness and rigor of the research (Henry, 2015; Johnson et al., 2020). Bracketing is a process whereby previous knowledge, beliefs, and common understandings about a phenomenon are set aside; this was done through an analytical and reflexive introspective review of the partnering university faculty’s emotions, perceptions, and reactions to the data with her research team during meetings (Tufford & Newman, 2012). Peer review is a process of working with a peer to discuss the interpretations from the data to allow for verification by another person. Faculty at the partnering universities maintained study memos that methodically described the step-by-step processes and decision-making; these were reviewed by a peer as a check on researcher bias (Johnson et al., 2020).

## Results

An overview of Goals 1, 2, and 3, data sources, and key findings can be found in Table 1.

**Table 1:** Overview of Data Sources, Analytic Approaches, and Key Findings by Goal

	Goal 1	Goal 2	Goal 3	
Goal	Illustrate how challenges were overcome in establishing an RPP to develop an equity-focused SEL program during the dual pandemic of COVID-19 and racial inequity in the early 2020s	Demonstrate how the RPP collaborated to co-develop the content of the lessons, engagement strategies, and implementation supports and present the resulting intervention	Assess the contextual appropriateness, acceptability, and feasibility of the RPP-developed program content and supports from the perspective of teachers and students	
Data Sources	Research team correspondence with districts and meeting minutes	RPP meeting agendas and minutes, correspondence, coach notes	Qualitative data collected through teacher semi-structured “exit” interviews, student focus groups	
Analytic Approach	Document review and synthesis by university and district partners	Document review and synthesis by university and district partners	Teacher Data Qualitative content analysis (multiple coders)	Student Data Qualitative content analysis (single coder)
Key Finding 1	Being intentional about disrupting white supremacy culture in educational research - Shifting away from a sense of urgency and focus on outcome - Embracing a focus on inclusive process and prioritization of building trusting relationships by slowing down	Following design-based principles and close teamwork, with working meetings over a year: - 4 × two-hours—student-facing elementary content review - 9 × two-hours student-facing middle school content review - 4 × two-hours teacher-facing content review	Successes reported by teachers included greater awareness of their own cultural norms, greater perceived student comfort with R-CITY lessons versus others, and feeling supported with planning and forethought to sensitive issues in lessons	Collaborative activities in the lessons were useful to ensure comprehension and clarity of complex vocabulary terms (e.g., “*ally*”) and concepts (e.g., “*empowerment*”), particularly for younger students
Key Finding 2	Fostering trust through a service-oriented alignment with district priorities - Project specific changes prior to launch (see Fig. 1) - Supporting district Equity Leads - Supporting schools the district prioritized for equity initiatives	Examples of RPP discussions to address concerns and develop solutions included: - Messages communicated by certain choices (e.g., microaggressions, privilege) - Solutions developed helped move beyond divisive discourse	Challenges reported by teachers were discomfort talking about racism, lack of representation of all student identities in lessons, sensitivity of topics, pushback from parents, and time required	Teachers need to attune to how demographic diversity (or lack of it) in the classroom composition can impact how the content is received by students during discussion
Key Finding 3	Enacting policy and regulatory protections for educational equity - District partners gained approval for 13 equity regulations by school board at optimal time	R-CITY multi-component equity-focused SEL intervention included - Grade-differentiated (4-5th, 6th, and 7-8th) student lessons - Teacher-facing implementation support materials - Structured coaching model and resources	Improvements suggested by teachers were to destigmatize coaching, systematize school-wide implementation supports, and adjust for heterogeneity within grade-level (developmental, ESL)	Engaging a diversity of youth to be directly involved in co-creation of content may help to ensure that all students can experience the lessons as authentic to them

SEL refers to social and emotional learning. RPP refers to research-practice partnership. R-CITY refers to the *Reducing Racism and Violence: Collaborative Intervention with Teachers and Youth* project. ESL refers to learning English as a second language

### Goal 1: How Challenges Were Overcome to Form an RPP for Equity in Early 2020s

In our document review, we identified three key ways we were able to establish our RPP: (1) Disrupting White Supremacy Culture; (2) Fostering Trust through a Service-Oriented Alignment with District Priorities; and (3) Enacting Policy and Regulatory Protections for Educational Equity.

#### Disrupting White Supremacy Culture

The first theme identified was the need to disrupt an aspect of white supremacy culture often inherent in applied, experimental school-based research, which is a tendency toward a sense of urgency and a prioritization of immediate results over an inclusive and healthy process, which can often take more time than expected (Okun, 2021). This theme is evident in an example of the originally planned partnership for this project, which dissolved in the winter of 2019. Specifically, as part of the project’s grant award process, the university partners engaged a district that had originally participated in the development of the proposal and provided a letter of support for the project (as it built on two prior projects at the middle and high school levels), and continued planning conversations with them in person meetings, calls, and emails through the fall of 2019. However, in December of 2019, the district communicated that it had decided not to collaborate on the proposed project due to misalignment with their current “direction of and priorities for work in social–emotional learning or racial equity.” This was during a time of nationally increasing accountability for racial equity, which was and continues to be an especially emotionally charged and contentious topic, and the district’s newly hired equity director was under tremendous pressure. These sensitivities certainly were in play in the dissolution of the partnership, however, the university partners also reflected that, in retrospect, they might have slowed down their approach to brokering a partnership with the district on the project with greater attention to process and inclusivity. For example, the university partners reflected that they may have been overly focused on asserting the aims of their research, when more lead time may have been needed to learn and understand the district’s broader equity and SEL-related goals. Relatedly, the university partner also recognized slowing down to focus on process could have provided a stronger foundation for communication. For example, the university partner’s vision for the intervention curriculum was to serve as a forum for students to receive support from their teachers related to their experiences of bias, with structured teacher professional development and supports. However, the district voiced concerns that an equity-focused SEL curriculum could send the message that the equity “work” falls on the shoulders of students, rather than the adults in the building. The university partners reflected that these gaps in perspectives on the work may have been bridged by intentionally fostering a slower process that emphasized opportunities for everyone’s voices to be heard (Okun, 2021).

This experience led the university partners to engage in a critical self-reflection process to clarify their values in focusing on process over outcomes as essential to meaningful and ultimately successful racial equity work in schools. They considered seriously the challenges of weaving together a project that met obligations to the funders, balanced the related requirements of what is deemed rigorous empirical research, and spoke to the priorities, assets, growth areas, and ongoing initiatives of potential partners. The outcome of this reflection process was a shift in orientation to be much nimbler and more service-focused in their next effort to connect with a district partner.

#### Fostering Trust Through Service-Oriented Alignment with District Priorities

A second theme from our document review highlights the importance of building trust as a foundation for partnership by ensuring priorities are aligned and demonstrating that alignment by making shifts in the project and taking a service-orientation to support district priorities. This theme was evident in the way the university partner approached their next effort to connect with a district partner. The university partner reached out to colleagues in a district with whom they had a long-standing collaborative relationship, with the idea in mind that their RPP could re-conceive of the project together, while attending to requirements of the funding mechanism. The approach taken drew on principles of RPP effectiveness, including a focus on building trust through a focus on relationships and supporting the practice organization (i.e., the district in this RPP) in accomplishing its mission and priorities (Henrick et al., 2017).

To kick-off the partnership, university faculty and coaches and the district partner’s equity office staff co-attended a practitioner-focused national conference called the *National School Board Association*’s Equity Symposium on February 1, 2020. Co-attending a non-researcher-oriented conference that the district partners valued provided an opportunity to get to know each other better and to engage in collective learning and discussion about equity initiatives occurring in the partner district and in districts nationally. This collective learning approach set a tone for the collaboration beyond the conference, and subsequent meetings with the chief equity officer and her team focused on understanding the context, goals, and priorities of the district’s equity office. As a result of this time spent listening and learning, it became clear that significant shifts in the originally proposed project to align with the district’s ongoing equity and SEL initiatives and priorities would be instrumental to the continuation of a true partnership.

The types of adjustments that the RPP made to the project were substantial. Specifically, the district’s equity office had been developing its own equity initiatives consistent with a theoretical model of equity literacy by Gorski (2016) that they had already adopted, with which the RPP agreed any racism and discrimination prevention elements of the project would align. Of note, the district view on equity was focused on equity for *all* students; as such, at this time, there was a concern that a focus on *racial* equity only was exclusionary and not aligned with their mission. For this reason, the RPP expanded the project’s focus to attend to equity based on gender identity, sexual orientation, (dis)ability, living on low income, religion, and other aspects of identity, while still centering racial equity within this broader frame.

**Fig. 1:**
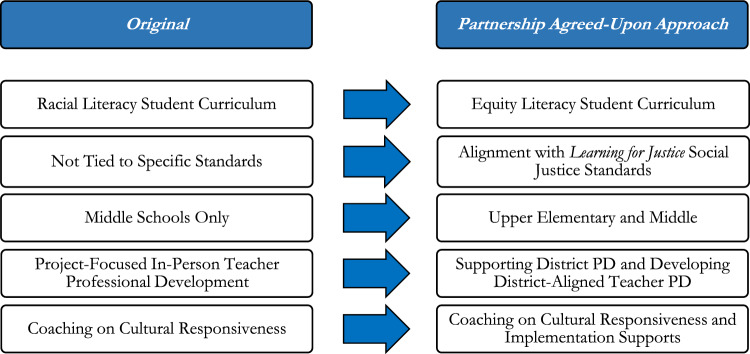
Re-Alignments with District Partner Priorities. *PD*  Professional Development

The project was also initially only focused on middle schools, but the district wanted to ensure that our reach included elementary grade levels, so we expanded our developmental focus to include 4th-8th graders. In addition, the district partner utilized the Learning for Justice (2022) *Social Justice Standards*, a set of 20 specific learning standards with accompanying grade-level differentiated learning outcomes, to develop their other equity-related content. The standards were anchored to four core social justice learning domains: identity, diversity, justice, and action, as part of their organizing framework for K-12 anti-bias education. As such, the RPP decided to align the lessons with the district’s broader equity initiatives and the standards from *Learning for Justice*. The RPP also decided to develop lessons to build teachers’ and students’ e*quity stamina*, a concept developed by the district partner that referred to both individual and group collective capacity to engage in equity-related work and conversations, even as they bring up difficult emotions and cause discomfort. Related to the university partners’ efforts to align with the district’s ongoing equity initiatives, the RPP also agreed to focus the university partner’s coaches’ efforts more on supporting their district-wide professional development and ensuring that the professional development offered through this project aligned with their foci. Through the RPP’s collaborative discussions at this stage, they also agreed upon the importance of ensuring that participating teachers were well-prepared to implement the lessons. Thus, coaching would be focused on providing specific, tailored implementation support related to challenges that may arise during the lessons to ensure no harm was done in their teachers’ coverage of topics to do with equity in the classroom. As the RPP agreed on these changes, which are summarized in Fig. 1, they decided to proceed with the project. In the university partner’s internal team meeting notes from February 20, 2020, they wrote at the top “[Redacted District] is IN! Working WITH the district, instead of asserting our [own plan] made THE difference!”.

Once the project launched, another key element of the success of the RPP’s collaboration was the service-focused alignment of the university partner’s coaches with district equity infrastructure, initiatives, and priorities. Within every school in the district (i.e., 120 schools), the district assigned 1–2 designated Equity Leads, teachers within the schools who were recognized formally in this role whose responsibilities included leading school equity team meetings and leading four annual, district-developed equity professional development trainings. These district professional development trainings were based on a year-long study of the book *Culturally Responsive Teaching and the Brain* by Hammond (2014) and occurred within schools quarterly on early dismissal days. The district engaged their Equity Leads in a separate train-the-trainer session, and as part of the RPP, the university partner’s coaches and project director attended relevant components of the train-the-trainer daylong trainings. The university partner’s coaches would then work with each of their school’s Equity Leads to provide differentiated support based on school and team needs as well as current resources. Support included attending and participating in school equity team meetings, co-planning the professional development trainings, and/or co-facilitating the professional development trainings.

Another aspect of the university partner’s service focus, in alignment with district priorities, was strategically engaging schools that were already participating in district initiatives to advance equity. Specifically, during the project the district launched or revamped initiatives (e.g., Black Boys Mentoring, Academic Achievement for All), for which schools had to apply to participate through a request for proposal process. Schools had to identify opportunity gaps in student achievement data and propose innovative programming to realize academic parity. To complement this work, schools participating in these initiatives were especially invited to participate in the project. As a reflection of the degree of recognition this project support received with the district and schools, the university partner’s coaches were invited as guests on a district program that is publicly available on the district’s own TV channel as well as on YouTube called *Open for Business*. This show highlights partnerships between the district and community partners on a monthly basis.

#### Enacting Policy and Regulatory Protections for Educational Equity Initiatives

The third theme identified in our review of documentation for Goal 1 was the importance of the district putting a policy and accompanying regulations in place to protect their equity goals and initiatives, in the face of wide pendulum shifts in societal support and backlash during this period. Specifically, the timing of the agreement to partner on this project taking place in spring 2020 coincided with the movement for Black lives spiking nationally and globally, with civil rights uprisings swelling to heights not seen for decades, leading to shifts in mainstream public support for racial justice and Black humanity in the near-term. With the sea change in sociopolitical support for racial equity initiatives that followed, and the rapid pace of transformation in the space of educational equity in 2020, the RPP has reflected over the years that this had been a critical moment to lock in protections for equity initiatives, in anticipation of the impending “whitelash” ahead.

A key example of this was when, in May 2020, district leadership in the equity office advanced and gained support to enact the equity policy they had been developing, consistent with a state educational policy passed in 2019 (COMAR 13A.01.06; COMAR 2021). The timing of the pandemic and George Floyd’s murder lent itself to the board unanimously approving the equity policy. The regulations stated that, “to achieve educational equity, all [redacted] district level offices will individually and collectively work to” accomplish 13 specific actions, which included attention to equity, access, and opportunity in: fiscal allocations of resources; social environments of schools; opportunities and access to resources for learning; professional development in cultural responsiveness; workforce diversification; cultural responsiveness in pedagogy, curricula, and instructional materials; strategic partnership building with external constituencies; strategic initiatives implemented to include implementation of equitable and culturally competent procedures and practices in each school; disaggregation and use of data to identify gaps and develop solutions; and methods for evaluating the impacts of their equity initiatives (Redacted, 2020). The passage of the district’s equity policy and accompanying regulations laid a foundation for an expansion of the equity office’s work. The district furthered its commitment to equity with these protections in place by approving a five-year strategic plan anchored in equity. This regulatory and policy protection made district support for continued partnership on the R-CITY project possible, despite later backlash and resistance. Importantly, the (albeit temporary) increase in public support for racial justice work in schools and passage of the equity policy in the district also made it possible for the RPP to fully embrace the call of the original funding mechanism to explicitly emphasize anti-racism as well as anti-bias more broadly. Although there was no guarantee that the policy would not later be overturned with changing public opinion, a majority of school board members’ votes would have been required to accomplish this, and state educational policy protected against it. Summoning the political will of a board to reverse itself on a policy they enacted to benefit all students would have taken a concerted effort and never transpired. As such, the district’s equity policy and regulations have continued to provide an important “cover” to pursue initiatives to advance equity without (or with less) fear of being attacked.

### Goal 2: RPP Co-Development and Finalization of the Intervention

For Goal 2, we describe the approach that we took to co-create content, provide examples of the types of revisions made to address district perspectives on contextual appropriateness, and briefly outline the multi-component R-CITY intervention that resulted from this process.

#### Approach to Co-Creating the Elementary and Middle School Preventive Intervention

To develop intervention content, the RPP followed design-based research principles (Anderson & Shattuck, 2012; Barab, 2014), which involved co-developing objectives with input from both researchers and practitioners, collaborative implementation, and working in partnership to interpret feedback and make decisions (Mulvey et al., 2020). Consistent with this approach, the RPP co-developed intervention materials and implementation supports and implemented them in the district partner’s schools. The RPP worked together to review intervention content, revise materials to enhance sensitivity to context and optimize acceptability and feasibility, implement the intervention, and assess teacher and student perspectives on acceptability.

To facilitate a collaborative teamwork process, the RPP reviewed iteratively developed drafts of content through a series of two-hour long working meetings. At each working meeting, district partners, including the chief equity officer and senior manager for equity, provided specific feedback on content and in-depth discussions ensued about key considerations in communicating with both teachers and students on various equity topics. At subsequent meetings, the revisions to the drafts made based on feedback from prior sessions were reviewed and finalized. Between January and March 2022, the RPP held four such working meetings covering content for Lessons 1–6 for the elementary iteration of the content. Between March and April 2022, they held another four working meetings focused on the teacher professional development content, including the creation of an accompanying equity glossary for teachers. Thereafter, between September 2022 and January 2023, they held nine working meetings, covering content for Lessons 1–6 for the middle school iteration of the content. Other meetings were also held during this time related to project implementation and data collection for the project; the meetings noted here were solely focused on the RPP’s iterative, collaborative intervention development process.

#### Examples of RPP Revisions to Optimize Contextual Appropriateness of Student Curriculum

The following are several examples of the types of feedback offered by district partners to ensure the contextual appropriateness, acceptability, and alignment of the content with the district’s approach to equity and local context of their county. One point of discussion was the use of the term *microaggressions*. The minutes from the meeting agenda read, “Are microaggressions really micro? Is this letting people off the hook? Need to focus on impact not intention.” The concern was about whether the term *microaggressions* communicated a message to teachers that minimized the magnitude of harm caused by implicit or covert forms of bias. This led to discussion of white fragility among teachers, and frustration with the possibility that the ‘micro’ in ‘microaggressions’ may be intended to mitigate the discomfort of White people in talking about interpersonal racism. The outcome was to include broader language around implicit bias with examples that highlight the degree of the impact of such encounters individually and over time, underscoring the need for accountability for impact, rather than intent.

In another example, district partners raised a concern, similar to the previously described prior district’s concern, that there may be an implied message communicated to students through the lessons about a responsibility being on their shoulders to cope with bias incidents. The notes from feedback related to this say:

*Need to tell kids to report it to a trusted adult. What is happening to them is not ok, it is unacceptable. The other person is the problem here. While we want you [students] to have these strategies, the adults need to make clear this is not acceptable, and it is not endorsed. This is not about taking ownership for what this other person is doing, but about loving ourselves up and getting ourselves care we need. But need to underscore a next step is telling a trusted adult*.

The outcome of this conversation was to ensure that the teacher lesson plans and coach-based implementation supports incorporated a dedicated focus on ensuring teachers communicate to students their responsibility and availability to provide support when incidents of bias occur. The district partners also highlighted that this would be consistent with their district’s policy on biased language and behavior. The RPP agreed that though there was some risk of misinterpretation, the intent and potential of the curriculum were to provide repeated, structured opportunities for teachers to foster open channels for communication. The set of “lessons” aimed to provide a regular forum for teachers to receive needed supports so they could in turn cultivate a space *with* students where students could feel safe and empowered to talk about experiences of bias, recognize them fully through mutual sharing and discussion, and find meaningful ways forward.

In another example, some lesson content initially used the word *privilege*, which the district partner identified as a ‘trigger’ word for some parents and teachers that could lead them to resist or disengage entirely from participation. This led to an in-depth discussion again about how to handle the issue of white fragility, with one perspective being a concern that adjustments may be accommodating fragility and another perspective that adjustments may be needed to meet participants where they are and engage them in the change process. These perspectives were both shared and understood by district and university partners. Though this discussion initially presented as a bind, continued discussion led to the deeper issue of *power*, including what power really was, and who held power in schools and in society, and why. This process led the RPP to develop a concept of power defined as not good or bad, but as having impacts could be harmful or beneficial depending on how power was wielded. As a result, the RPP entirely reworked Lesson 1 for 7th–8th graders to be called *Finding Your Power*, with a focus on helping students to define power and teaching differences between ‘power over,’ ‘power with,’ ‘power within,’ and ‘power to,’ consistent with a model of power by Brown (2023). This was a profound shift in the way they were approaching the content that was much better aligned with their goal of supporting young people’s empowerment in the face of bias. Further, the process was revelatory of the potency of the collaborative teamwork they were engaged in to uncover new ways of approaching equity work that could be unifying and empowering, rather than stuck in a win-lose binary view. This example, and others like it, also helped the RPP to see clearly how nuanced and complex equity topics are, which affirmed their decision to provide extensive coaching support for teachers, a glossary of terms, and professional development to support teachers’ implementation of the lessons.

#### The R-CITY Preventive Intervention for Elementary and Middle Schools

After two years of collaboration to develop and implement the R-CITY intervention, the RPP completed a multi-component, equity-focused SEL intervention for upper elementary to middle school grade levels (4^th^–8th), including three components: (1) a student curriculum, (2) a set of implementation supports, and (3) a structured coaching model and materials.

##### Student Curriculum

The teacher-led, equity-focused SEL curricula featured three grade-differentiated sets of six lessons for upper elementary and middle schoolers (upper elementary 4-5th, transition to middle school 6th, and upper middle 7-8th). Each lesson was designed to be 30 min long and to be implemented during students’ advisory (homeroom) period in the morning, once per week. Equity-focused SEL lessons included some topics which with transformative SEL (Jagers et al., 2018), such as understanding *identity* and how it relates to *belonging*, finding your power (i.e., *agency*), learning about histories of injustice (i.e., *curiosity*), and speaking up to bias and equity action planning (i.e., *collaborative problem-solving*). Lessons also focused on the importance of self-care and self-compassion in the context of experiencing bias and injustice. Lessons were organized to include a *Warm-Up* to spark student engagement and review previous lessons; an *Introduction* to introduce new lesson concepts and vocabulary; a *Discussion and Activity* featuring small group, partner work, or individual processing, with videos, discussion questions, and handouts; and a *Wrap Up* with guided questions to check for student understanding. Lessons were organized in a teacher-accessible Google Drive.

##### Implementation Supports

A binder was provided during 60–90 min training sessions in participating schools, delivered by the project coaches. The binder included 2-page teacher lesson plans and copies of the student handouts (sufficient for 25 students) for each lesson, as well as other resources like a glossary of terms. The training covered the purpose of the project, the goals of the lessons overall and objectives of each lesson, the challenges that may arise in implementation and recommended solutions, and resources and tools for implementation planning, including a lesson schedule for teachers to tailor in partnership with coaches based on their timeline for implementation. The lesson plans included teacher scripts and sections for *Objectives*, *Materials and Resources*, *Vocabulary*, and *Engagement Tips*. The latter section highlighted relevant context, concerns with implementation that might arise, and strategies for sensitive engagement for each lesson topic, consistent with the training foci.

##### Structured Coaching Model

The R-CITY coaching model employed coaches from the research team, utilized motivational interviewing (Miller & Rollnick, 2023), and was intentionally non-evaluative. The model provided one-on-one coach supports to teachers through a step-by-step approach to help teachers apply culturally sustaining classroom practices and support their equity stamina. The coaching also aimed to improve implementation quality of the teacher-led, equity-focused SEL curricula. Steps were (1) help teachers reconnect with their values; (2) evoke teacher-led goals; (3) map evidence-based, specific strategies to those goals using an evidence-based strategy map resource; (4) plan strategy implementation, and (5) observe or provide feedback on implementation. Five, 45-min coach-recorded webinars were also co-developed and available on: (1) Building authentic relationships, (2) Sensitivity to students’ identities, (3) Understanding racial injustice and its impacts on students, (4) Talking about racial equity in the classroom, and (5) Strategies for critical self-reflection.

### Goal 3: How the Intervention was Received by Teachers and Students

For Goal 3, we report two sets of results of analyses of these data. Teacher findings focused on challenges, successes, and suggested improvements. Student themes specified the utility of the collaborative activities, attunement to how demographic composition of the classroom can impact how the content is received, and the importance of ensuring that students experience the lessons as authentic.

#### Teacher “Exit” Interviews Results

The teacher semi-structured interviews revealed successes, challenges, and suggested improvements for the R-CITY intervention. For successes, teachers appreciated that preparing for the lessons was not time-consuming and that the lesson training was simple and direct. As a result of training, teachers reported feeling more aware of their own cultural norms and the need to understand both their own and their students' cultures. Teachers also stated that they believed students were more open and comfortable with the equity-focused SEL lessons than with other SEL lessons. They described feeling supported with planning and giving forethought for what might trigger certain students.

For challenges, teachers noted some difficulties talking about cultures other than their own, at times mentioning a lack of experience with those cultures. Specifically, some teachers reported feeling that it was challenging talking about race and racism in the classroom, both because of their own discomfort as well as trauma-related discomfort that arose among their students. In addition, the teachers expressed that the lessons did not always represent every identity in their classroom. This was challenging for them when they felt they needed to speak to those identities that were not represented in the lessons, but did not feel like they knew enough about students’ identity backgrounds to do so. For example, one teacher said, “I think race is an uncomfortable topic to talk about when you're in a room with all different races. You don't know. You want to make sure that you're not hurting anyone's feelings, or you're saying something that's going to offend them. And you don't know what their knowledge is or their background so…sometimes those students have a sensitivity that I wasn't even aware of.” Teachers reported fearing misspeaking or that there would be some misrepresentations on their part.

In addition, teachers sometimes reported that students became less engaged when it seemed the topic was too personal. In these cases, the teachers noted that trauma issues may have come up and that they needed to make thoughtful adjustments to support students and ensure the lesson content was not triggering. Another teacher reported a challenge that students were always using the word ‘racist,’ not fully comprehending what it meant. “My sixth [grade] readers [students] call each other racist. All day long. I don't think they really understand what that word means. I don't think they know the definition, or they know what it looks like, or they know what it sounds like. It's something that needs to be taught to them.” Though a challenge, this also underscored the need for the lessons.

Teachers also reported dealing with parent push back. Specifically, one teacher shared, “I had a parent contact me like, ‘Why are you bringing in the word racist into your classroom?’ and she CC'd my principal.” Teachers reported parent push back about the SEL aspect as well and indicated a need for guidance for how to handle it. As another teacher noted, “I feel like, as teachers, we need specific language and specific things that we need to say. …I mean, we do have parents that reach out and they do not want their child participating in those lessons. …Or being around those conversations.” Lastly, teachers frequently commented that time is a significant barrier and challenge to implementing the lessons and coaching. One teacher felt that R-CITY content should be provided through a dedicated, regular, yearlong class, and not as an add-on, just during a small segment of the day during one time of the year.

For suggested improvements, the teachers who participated in exit interviews recommended re-framing the “coaching” as consultation, as the term coaching might be stigmatizing and discourage teacher participation. In addition, teachers often suggested that school-wide supports for implementation were needed, such as incentives like having other classes covered for those delivering the equity-focused lessons. Regarding the lessons themselves, teachers sometimes felt that the content could be further developmentally tailored (less advanced) for elementary grades 4–5. This was an area the RPP felt challenged by, as the partners tried to attend sensitively to presenting equity vocabulary and concepts that were developmentally appropriate at each stage, yet even more attention to this appeared to be needed in part because students were behind normative reading levels following COVID-19-related pandemic closures. In addition, teachers asked for more models of lesson implementation, such as through videos that showed well-implemented lessons. Some teachers commented that the lessons were less accessible to their students who were learning English as a second language, and suggested word banks and separate handouts might help them follow along better. Last, teachers felt that more guidelines for difficult conversations were needed at the beginning of the lessons. Although the RPP was careful to include lesson-specific guidelines, content warnings, teacher tips, and coaching supports, teachers still often wanted even more support. Teachers also felt that expanded warm-ups were needed in some cases to help students approach harder ideas and concepts in the lessons.

#### Student Focus Groups Results

Three themes emerged from focus group data collected from students about the content of the equity-focused SEL lessons. The first theme was that collaborative activities promoted engagement and helped facilitate clarity. Focus group participants found that small group, partner work, and discussion questions were *most* engaging due to their collaborative nature. These lesson activities were often noted as a favorite part of the lesson. When complex vocabulary terms (e.g., “*ally*”) or concepts (e.g., “*empowerment*”) were used in lessons, younger adolescents spoke to the utility of collaborative activities for comprehension and clarity, even though they may have lived the experience. An 11-year-old stated, “I was confused, but when I got to talk about it in the situation [i.e., handout] it helped me to better understand what was meant.”

The second theme was that discussion must support belonging and not promote isolation*.* Though collaborative activities were reported as engaging, focus group participants cautioned that teachers must facilitate discussion with great care. Several focus group participants highlighted that while they wanted their opinions to be heard and valued by the class, they did not want discussion opportunities to make them feel singled out as the “diversity” expert or representative. This theme was especially prevalent for focus group participants who noted that due to their racial group membership they were often the numerical minority in their classrooms. A 13-year-old youth noted: “I just don’t like when the whole class be looking at me, cause like my school is not that diverse. So sometimes I just be in the front, and I'll look behind me and everybody looking at me when we talk about this and then you just like get singled out.”

The third theme was that lesson content should be authentic*.* Even with the need to take great care, students appreciated the real-world racial equity topics and examples discussed in equity-focused SEL lessons. An 11-year-old focus group participant noted, “I like how [the lesson] talked about the different problems that our generation has and some ways to help. It seemed to get at real stuff.” In contrast, students called out content that they felt was inauthentic as unrelatable. For example, a 14-year-old participant noted the same video included outdated content: “Those are like the old-fashioned stereotypes of different groups and people… like there's different ones you can talk about now. Not just those kind of ones.” The RPP observed this contrast may have been in part related to student age and developmental stage, where older students (i.e., at or near the transition to high school) were able to discern and observe more complex and nuanced forms of interpersonal bias, and thus wanted examples that were more relatable to their experience. Similarly, another 14-year-old focus group participant noted, “I felt like the video was just a little bit too cheesy… it was just a little bit much with the music, too. It's a little bit too much.” Interestingly, this comment was in reference to a video created by youth from the district and edited by adult staff that the RPP had incorporated into the middle school lessons as a way of ensuring local contextual appropriateness and relevance of the content. The RPP observed that, even with such a close level of local integration, content still may not resonate with all youth participants, suggesting the need for a diversity of youth to be more directly involved in leadership and co-creation to ensure its relevance.

## Discussion

This study described a university-district RPP to develop a multi-component, equity-focused SEL preventive intervention, inclusive of grade-level differentiated, teacher-led universal classroom SEL curricula. In addition, the multi-component intervention included a series of implementation supports, adult coaching, and professional development for teachers. This work leveraged the strengths, talents, and expertise of a multidisciplinary university-district RPP. The ultimate goal of the project was to extend adult and student SEL skills to promote equity and prevent violence in elementary and middle schools.

### Lessons Learned

During a window in 2020 of greater accountability nationally for racial justice, many educational institutions were more open to elevating initiatives focused on diversity, equity, inclusion, and justice. A key lesson learned was that enacting policy and regulations to structurally support schools’ equity work can reap many benefits, including protection against a backlash period. The district’s seizing this opportunity provided a protective buffer for the R-CITY-focused work of our RPP. As a result of these protections, the RPP was able to closely collaborate and prioritize time for honest dialog on equity topics and ‘in the weeds’ conversations necessary to develop and refine the intervention, as well as cultivate readiness for its implementation. The RPP was also able to identify specific contextual modifications and considerations needed to navigate the pushback on equity and SEL, including evolving restrictions on what teachers can say about identity, in ways that still advanced the work of equity in schools.

The close teamwork aspect of the RPP on the co-development of the student- and teacher-facing equity intervention ultimately became the “heartbeat” of the partnership; through this teamwork, district and university partners spent time together, shared personal stories, were vulnerable and human with one another. The teamwork in this space allowed the partners to build trust, attune to one another’s theoretical principles and visions driving the equity content, and share communication and messaging strategies to navigate sociopolitical backlash on SEL and equity.

Another lesson learned was the importance of focusing on equity-focused SEL knowledge and skill among teachers and other adults at school. Adult skills in equity-focused SEL cannot be assumed, and attention to building adult skills should predicate implementation of student-facing interventions focused on anti-bias skills. Consistent with motivational interviewing core principles and practices (Miller & Rollnick, 2023, also see Herman et al., 2021), R-CITY emphasized the need to “come alongside teachers” (p. 239) and work with their values to elicit their own change talk, rather than conveying to teachers that they need to change. Although accountability absolutely holds a vital role in advancing anti-racism and equity in schools, that accountability must be situated in the context of trusting relationships in which teachers’ autonomy and capabilities are respected, to facilitate the types of changes that are needed. Often accountability initiatives related to discipline disproportionality, for example, are conveyed in top-down ways that elicit feelings of fear, shame, and threat among teachers, which is counter-productive to the organizational goals for change. The coaching approach adopted by R-CITY emphasized meeting teachers where they were and focusing on their strengths to motivate growth. Importantly, the coaching approach taken in the R-CITY intervention also provided therapeutic, occupational wellbeing-focused supports for teachers.

The importance of carefully considering developmental windows for young people to learn about anti-racist and culturally sustaining content, and tailoring interventions appropriately, was another lesson learned. Based on feedback from teachers and students, the RPP considered the fairly wide-ranging variation in students’ capacities to engage analytically and abstractly with equity concepts from upper elementary to the transition to high school. In addition, the student feedback suggested that students’ sensibilities about equity issues become more nuanced at the transition to high school.

Relatedly, by focusing on understanding student perspectives through student focus groups, the RPP found that some components of the adult-created R-CITY lessons did not necessarily have the intended meaningful impact for students as expected. Through feedback from students, the RPP was able to see firsthand the value of centering youth’s “organic intellectualism” and recognizing young people as experts who are most affected by educational and social inequities (Newman et al., 2021; Noltemeyer & Grapin, 2021). This work requires honestly and intentionally grappling with our country’s collective racist and oppressive history, including that within the education system (Anderson et al., 2019; Saleem et al., 2022; Strunk & Andrzejewski, 2023). Youth possess direct experiences and insights into the challenges faced and practices needed to support their learning and development. Though adolescents demonstrate a growing capacity and desire to exercise control over issues impacting their lives, including ways to address racial equity, young people perceive fewer opportunities to exercise autonomy and participate in making practice decisions (Ozer & Wright, 2012; Rowan et al., 2024). Efforts toward social justice in education are propelled by the experiences of those who have crucial insider knowledge about systemic educational inequity (Cammarota & Romero, 2009; Rodríguez & Brown, 2009). Yet youth of color are often intentionally and systematically excluded from adult decision-making spaces and their voices are often effectively silenced as they are not granted authentic and meaningful decision-making opportunities that impact school practices.

These reflections led the RPP to consider the need to elevate students as drivers and developers of the intervention as a future direction, through the inclusion of youth participatory action research. The RPP realized that centering the voices of students would not simply be an additional value, but critical when co-creating student-facing social–emotional learning content that is engaging, authentic, and supports belonging. In tandem with developmental reflections on the transition to high school noted above, the RPP decided to plan for a high school iteration of the equity-focused SEL preventive intervention that would further center student voice by integrating a youth participatory action research element (Bettencourt, 2020). The RPP is pilot testing the incorporation of four youth participatory action research focused lessons in the student curriculum through a separate seed project funded by the William T. Grant Foundation (PI: Brown Griffin). This pilot work is an extension of the RPP’s evaluation of the 4th-8th grade R-CITY equity-focused student curriculum and teacher-facing implementation and coaching supports through an ongoing randomized controlled trial (Project Director: Bottiani, PI: Bradshaw). The RPP will leverage findings from these evaluations to begin thoughtful, dedicated work in partnership with youth to develop the high school intervention.

Another future direction is that the district equity office would like to make available the R-CITY equity-focused SEL lessons for use district-wide as part of their four quarterly *Unity Day* lessons. To accomplish this, the RPP has discussed the development of an asynchronous train-the-trainer approach in collaboration with the district equity specialists and the school equity leads, potentially offered through their office of professional learning and organizational advancement to provide continuing education credits. This train-the-trainer approach could include training in the lessons as well as the coaching model to build capacity within schools to sustain the R-CITY research team coaching. Given the district-appointed equity leads’ role is to support school-level processes and teacher professional development to promote equity, training them would provide a natural transition from the research team coaches in our ongoing scale-up and sustainability efforts.

### Strengths and Limitations

A significant strength to note is the durability of the broader institutional partnerships between the research center in which this study was housed and the district, which spanned nearly two decades and provided the foundation for the RPP’s work. This partnership includes multiple researchers as well as several district personnel in different departments of the district. The broader RPP foci and goals have adapted and branched out over time across myriad initiatives to address emerging and ongoing needs identified within the district. The present study reports on just one initiative among these ongoing, interrelated activities.

On the other hand, a limitation of such close partnership can be that compromises are made, such as our work building a curriculum around a framework that did not yet have empirical data to support it (Learning for Justice, 2022). However, these choices to prioritize alignment in the service of the district’s goals were core to the success of the overall RPP. Another challenge was that in this iteration of the work, the RPP did not have the opportunity for rapid prototyping (i.e., iterative cycles of development, implementation, feedback, revision, etc.). Once the intervention content was developed, it was not further changed. Instead, the feedback the RPP collected is being channeled to create a high school model. Finally, the university partners faced challenges in achieving sustained teacher and school engagement amid ongoing COVID-19 disruptions and educator staffing shortages that were affecting districts nationally during this time.

## Conclusion

Integrating equity topics into SEL programming and culturally sustaining practices foci into teachers’ professional development supports are vital steps in the broader goals of reducing youth exposure to racism and discrimination and fostering school contexts supportive of mental health for young people. These types of initiatives are challenged by the sociopolitical climate and its vicissitudes, but strong RPPs are foundational to weathering these storms. This RPP was able to co-create a fully developed, multi-component program to promote changes in teacher classroom practices and instruction in upper elementary and middle schools with the goal of preventing bias exposure and ultimately violence. In the politically charged context during which this research was conducted, there was a need to consider the growing national and local potential for concerns related to SEL and equity-related programming held by parents and other constituencies (e.g., school board members, principals), which could adversely impact implementation or put educators in a vulnerable position as implementers. For these reasons, the research-practice partnership between the university and district partners became more important than ever to balance these needs and sensitivities when developing and implementing the equity-focused SEL intervention.
